# Computational Analysis of Gly482Ser Single-Nucleotide Polymorphism in PPARGC1A Gene Associated with CAD, NAFLD, T2DM, Obesity, Hypertension, and Metabolic Diseases

**DOI:** 10.1155/2021/5544233

**Published:** 2021-08-05

**Authors:** Somayye Taghvaei, Leila Saremi, Sepideh Babaniamansour

**Affiliations:** ^1^Department of Medical Biotechnology, National Institute of Genetic Engineering and Biotechnology, Tehran, Iran; ^2^Department of Biology, Science and Research Branch, Islamic Azad University, Tehran, Iran; ^3^School of Medicine, Islamic Azad University Tehran Faculty of Medicine, Tehran, Iran

## Abstract

Peroxisome proliferator-activated receptor-gamma coactivator 1-alpha (PPARGC1A) regulates the expression of energy metabolism's genes and mitochondrial biogenesis. The essential roles of PPARGC1A encouraged the researchers to assess the relation between metabolism-related diseases and its variants. To study Gly482Ser (+1564G/A) single-nucleotide polymorphism (SNP) after PPARGC1A modeling, we substitute Gly482 for Ser482. Stability prediction tools showed that this substitution decreases the stability of PPARGC1A or has a destabilizing effect on this protein. We then utilized molecular dynamics simulation of both the Gly482Ser variant and wild type of the PPARGC1A protein to analyze the structural changes and to reveal the conformational flexibility of the PPARGC1A protein. We observed loss flexibility in the RMSD plot of the Gly482Ser variant, which was further supported by a decrease in the SASA value in the Gly482Ser variant structure of PPARGC1A and an increase of H-bond with the increase of *β*-sheet and coil and decrease of turn in the DSSP plot of the Gly482Ser variant. Such alterations may significantly impact the structural conformation of the PPARGC1A protein, and it might also affect its function. It showed that the Gly482Ser variant affects the PPARGC1A structure and makes the backbone less flexible to move. In general, molecular dynamics simulation (MDS) showed more flexibility in the native PPARGC1A structure. Essential dynamics (ED) also revealed that the range of eigenvectors in the conformational space has lower extension of motion in the Gly482Ser variant compared with WT. The Gly482Ser variant also disrupts PPARGC1A interaction. Due to this single-nucleotide polymorphism in PPARGC1A, it became more rigid and might disarray the structural conformation and catalytic function of the protein and might also induce type 2 diabetes mellitus (T2DM), coronary artery disease (CAD), and nonalcoholic fatty liver disease (NAFLD). The results obtained from this study will assist wet lab research in expanding potent treatment on T2DM.

## 1. Introduction

Peroxisome proliferator-activated receptor-G coactivator 1-alpha (PPARGC1A, PGC-1*α*, or PGC-1) is a transcriptional coactivator of peroxisome proliferator-activated receptor gamma (PPAR-*γ*), which regulates the energy metabolism's genes and mitochondrial biogenesis [1, 2]. The nuclear receptor PPAR-*γ* enables PPARGC1A to interact with various transcription factors. PGC-1*α* also regulates the cAMP (cyclic adenosine monophosphate) response element-binding protein (CREB) and nuclear respiratory factors (NRFs). The PGC-1*α* protein is also associated with controlling blood pressure, cellular cholesterol homeostasis, and obesity [3, 4]. Thus, the PGC-1*α* encoding gene plays an essential role in cardiovascular and metabolic diseases. It also regulates the pathophysiological processes contributing to coronary artery disease (CAD) [5–7].

PGC-1*α* regulates the gene expression of mitochondrial fatty acid oxidation enzymes through interaction with peroxisome proliferator-activated receptor- (PPAR-) alpha in the heart, brown adipose tissue, and liver [8]. PGC-1*α* also increases glucose uptake in the muscles by regulating glucose transporter 4 [9]. In addition, it increases the gene expression of phosphoenolpyruvate carboxykinase and glucose-6-phosphatase, which is vital for hepatic gluconeogenesis [10]. These critical functions of PGC-1*α* in the regulation of adaptive cellular energy metabolism, vascular stasis, oxidative stress, and adipogenesis led to conducting a study on the relationship between PPARGC1A variation and a range of metabolism-related diseases [5].

Single-nucleotide polymorphisms (SNPs) are widely divided into two distinct clusters, synonymous (csSNPs) and nonsynonymous SNPs (nsSNPs) [11]. The nonsynonymous SNPs are further divided into missense mutations and nonsense mutations. The coding synonymous SNPs have a low effect over protein structure, while the nonsynonymous SNPs have a great impact on the protein structure and higher risk of diseases [12, 13].

Thus, they have particular importance for additional experimental assessment. In silico studies supply an efficient platform for analysis and evaluation of genetic mutations for their pathological consequence, and defining their underlying molecular mechanism [14–18]. The G to A substitution in exon 8 of the PGC-1a gene leads to the substitution of glycine with serine in codon 482 that reduces PGC-1a expression and PGC-1a protein activity [19].

In the present study, we surveyed the literature on the deleterious effect of SNP G>A Gly482Ser in the PPARGC1A protein coding region. Despite the controversial results of studies, many reports related the PPARGC1A gene's polymorphisms to type 2 diabetes mellitus (T2DM), obesity, and hypertension [20].

Gly482Ser (+1564G/A) polymorphism is one of the most widely studied. Gly482Ser is the most critical and common PPARGC1A gene SNPs, corresponding to a missense variant in the coding sequence [6, 21]. Frequency of this variant in the gnomAD database is 0.3 with 12728 homozygous and 77425 heterozygous. From the beginning, many of the studies have reported associations of Gly482Ser (+1564G/A) variation with diabetic complications [22–25]. We also investigated the effect of Gly482Ser polymorphism on nonalcoholic fatty liver disease (NAFLD) and the risk of coronary artery disease (CAD) among patients with T2DM [26–28]. We then used computational studied to further investigate this polymorphism.

After we modeled the structure of PPARGC1A protein, we substituted Gly482 with 482Ser and predicted the effect of Gly482Ser variant on the stability of PPARGC1A using prediction SNP tools. The molecular dynamics simulation (MDS) is a promising approach to examine the conformational changes in the Gly482Ser variant structure with respect to the native conformation [29–36]. Researches indicate that MDS can detect the changes in protein phenotype that significantly contribute towards confirming the damaging consequences of computationally predicted disease-associated mutations [16].

We focused on investigating that the changes in the dynamic behavior of PPARGC1A was induced by the pathogenic G>A Gly482Ser variant. Experimental studies have indicated that G>A Gly482Ser variant causes disease. We conducted MDS to reveal the conformational changes occurring in the Gly482Ser variant structure which may account for the observed molecular changes and the related pathological outcomes. The simulation also reveals the conformational flexibility of the Gly482Ser PPARGC1A variant to show how this variant affects the protein and pathogenesis of the related diseases. We also performed essential dynamics (ED) and molecular docking for the survey of this variant. In general, our results provide strong evidence of main conformational drift occurring in the Gly482Ser variant as compared to the native.

## 2. Materials and Methods

PPARGC1A sequence data was collected from the national center for biological information (NCBI) protein sequence database. rs8192678 (+1564G>A Gly482Ser) SNP information for our computational analysis was retrieved from the dbSNP database (http://www.ncbi.nlm.nih.gov/projects/SNP/, access date: February 30, 2019) [37]. The studies were performed in the RCSB PDB (https://www.rcsb.org/) [38] and UniProt (https://www.uniprot.org/uniprot/O75369) [39] databases to find the suitable crystallographic structure of the PGC-1*α* protein with ID Q9UBK2.

### 2.1. Modeling of Protein, Modeling Evaluation, and SNP Creation

After investigating RCSB PDB, the appropriate structure that includes the polymorphism site was not found so the structure of PGC-1*α* was modeled.

As communitywide blind CASP experiments have indicated which I-TASSER server can now create structural models with accuracy similar to the best human expert-guided modeling [40] and compared with other useful online structure prediction tools, the I-TASSER is in the reliability and notable accuracy of full-length structure prediction for protein targets with various difficulty and the wide structure-based function predictions [41]. Then, we selected I-TASSER (https://zhanglab.ccmb.med.umich.edu/I-TASSER/) [42] server for modeling the human PPARGC1A protein structure (with 798 aa). The quality of the modeled PPARGC1A protein structure was evaluated independently by the VADAR version 1.8 (http://vadar.wishartlab.com/). Since our study was on the Gly482Ser polymorphism, we replaced glycine residue of the wild-type (WT) protein to serine residue in the variant using the SPDB viewer [43]. The structures were minimized with YASARA program [44] and were applied to the study.

### 2.2. Stability Analysis Using SNP Tools

Since a missense polymorphism causes the alteration of the protein structure and function, therefore, we predicted protein stability. A number of recent studies have verified that implementing multiple bioinformatics tools and algorithms increases the accuracy of the results [45]. To evaluate the effect of the amino acid substitution at 482 position on the stability of wild-type PPARGC1A, we used the following stability predictor tools. MUpro is an assembly of programs with machine learning that computes the protein stability and changes based on sequence data, especially when the tertiary structure is not subjected. This approach dominates significant restrictions on previous approaches based on the tertiary structure [46]. The CUPSAT tool evaluates and predicts protein stability based on mutations [47]. DynaMut can perform rapid analysis of the protein stability and dynamics coming from alterations in vibrational entropy [48]. DUET also predicts the effect of point mutations on the protein stability through an embedded computational approach [49]. The mCSM calculates the consequences of missense polymorphisms on the stability of protein, protein-protein binding, and protein-DNA interaction [50]. SDM considers the amino acid substitutions of different structural conditions tolerated in the families of homologous proteins of specified 3D structures and converts them into possibility tables for amino acid substitution [51]. I-Mutant2.0 calculations are based on the protein structure or the protein sequence or are based on prediction of protein stability of missense variants [52]. PANTHER also predicts evolutionary evaluation of the coding SNPs [53]. To evaluate deleterious effect of the Gly482Ser variant on the interaction of the PPARGC1A protein, then to investigate the effect of the Gly482Ser variant on the PPARGC1A function and interaction, we performed molecular docking.

### 2.3. Protein-Protein Molecular Docking

Protein-protein interactions have a significant role in different cellular processes and are also involved in various diseases. They are also a highly significant target for therapeutic interventions [54]. PPARGC1A is a transcriptional coactivator of peroxisome proliferator-activated receptor gamma (PPAR-*γ*), which regulates the energy metabolism's genes and the mitochondrial biogenesis. The nuclear receptor PPAR-*γ* enables PPARGC1A to interact with various transcription factors [1, 2]. We employed ZDOCK (http://zdock.umassmed.edu/) to evaluate deleterious effect of the Gly482Ser variant on the interaction of the PPARGC1A protein with PPAR-*γ*. 292-403 amino acids from PPARGC1A were selected as PPAR-*γ* binding domain and 317, 351, 477, and 501 amino acids as interaction site of PPAR-*γ*. ZDOCK uses the fast Fourier transform algorithm for an efficient global docking on the 3D grid. ZDOCK also uses the combination of shape complementarity, electrostatic, and statistical potential for scoring the docked complex [55].

### 2.4. Molecular Dynamics Simulation

#### 2.4.1. MD Simulation

This study was performed using the basic tool of GROMACS [56]. MDS was carried out with the parallel version of PME in the GROMACS program. Each one of structures was immersed in a dodecahedron-modeled box (*x*, *y*, and *z*) with 238.58 nm^3^. SPC/E water molecules were used to solvate the system. The nonbonded cut off was set at 10 Å, and every 5 steps, the nonbonded pair list was updated. LINK mode was applied to constrain all hydrogen bonds and motion equation integration [57]. MDS of PPARGC1A was started through 1000 steps of energy minimization with solvation within a dodecahedron-shaped water cage with 1 Å of the distance between protein periphery and the cage edges. System neutralization was done by adding 15 NA ions. Molecular dynamics simulation was performed at 300 k (physiological temperature), pH = 7 using GROMACS 4.6.5 (http://www.gromacs.org/), and the GROMOS53a6 force field. Before the MDS run, the structures were gained to a temperature of 300 K and were equilibrated during 100 ps under constant volume and temperature (NVT). Next, the system was switched to continuous pressure and temperature (NPT) and equilibrated for 100 ps. All the periodic boundary condition functions were carried out using the leap-frog algorithm with a 2 fs time step, and every 500 steps, structural snapshots were flushed [56]. 50 ns MD simulations of the Gly482Ser variant and the native of PPARGC1A in 25 × 106 were steps individually done. The cutoff radius of protein-solvent intramolecular hydrogen bonds was 0.3 nm.

#### 2.4.2. Analysis of Molecular Dynamics Trajectories

Structural deviation analysis of the Gly482Ser variant and wild-type protein such as root-mean-square deviation (RMSD), root-mean-square fluctuation (RMSF), solvent accessible surface area, gyration radius, hydrogen bond, and the secondary structure of the protein (DSSP) was computed using g_rmsd, g_rmsf, g_sasa, g_gyrate, g_hbond, and do_dssp built-in functions of GROMACS package. GRACE software was used to plot graphs (http://plasma-gate.weizmann.ac.il/Grace/) [58].

### 2.5. Essential Dynamics

Essential dynamics, known as principal component analysis (PCA), can show the collective atomic motion of the wild-type and Gly482Ser variant proteins by the GROMACS tool [59]. Principal component analysis was computed using g_covar and g_anaeig built-in functions of GROMACS package. PCA is a standard protocol for the characterization of eigenvectors and the projection across the first PC1 and PC2 [60].

## 3. Results

### 3.1. Protein Modeling, Modeling Evaluation, and Replacement Gly to Ser at 482 Position in the PPARGC1A

**Figure 1:**
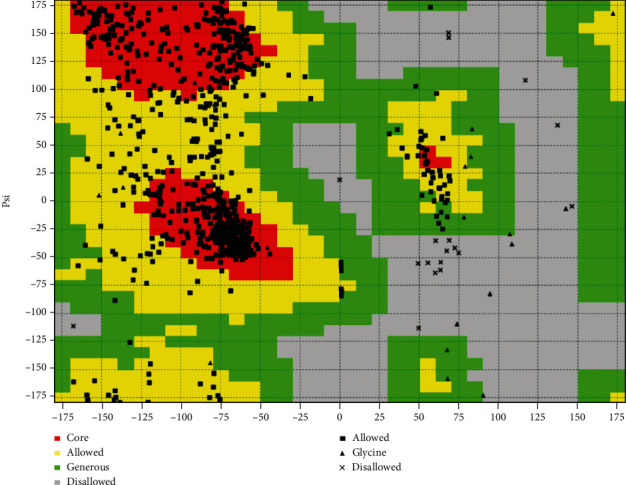
The Ramachandran plot in the evaluation of the 3D structure was modeled using VADAR server.

The modeling using I-TASSER gave five models. Model 3 with the highest C-score was selected for further studies. Modeling evaluation of Model 3 by VADAR server was showed 94% of the amino acids of the modeled structure in the allowed area (Figure 1), meaning that this model is suitable for further study. Amino acid replacement was also done using SPDB viewer. In the next step, the effect of Gly482Ser polymorphism on the structure and function of PPARGC1A was exhibited by SNP tools.

### 3.2. Stability Prediction with SNP Tools

It was demonstrated that most of the disease-associated polymorphisms have a significant influence on the protein stability. We used eight different computational prediction tools based on different algorithms to calculate the impact of the Gly482Ser variant on the PPARGC1A structure and function. Stability of the protein is often changed by SNPs. I-Mutant2.0 and MUpro reported that the Gly482Ser variant decreases stability while providing the DDG value (Table 1). PANTHER reported that the Gly482Ser variant is probably damaging. Results obtained from CUPSAT, DUET, DynaMut, mCSM, and SDM showed a destabilizing impact of the Gly482Ser variant (Table 1). DGG of CUPSAT and PANTHER were not provided. In the next step, we studied the effect of this variant on the interactions of PPARGC1A.

### 3.3. Protein-Protein Molecular Docking

We carried out molecular docking using a freely available ZDOCK online server that predicts protein-protein complexes based on rigid-body docking programs. We observed a ZDOCK score of 1847.281 for native PPARGC1A protein interaction with PPAR-*γ* protein compared with variant Gly482Ser with an interaction score of 1663.332. This finding further strengthened our theory that SNP Gly482Ser has deleterious effects on the structural and functional attributes of the PPARGC1A protein. At the next step, we simulated the Gly482Ser variant and native protein to explore conformational changes and the Gly482Ser variant stability compared with the native protein.

### 3.4. MD Simulation

**Figure 2:**
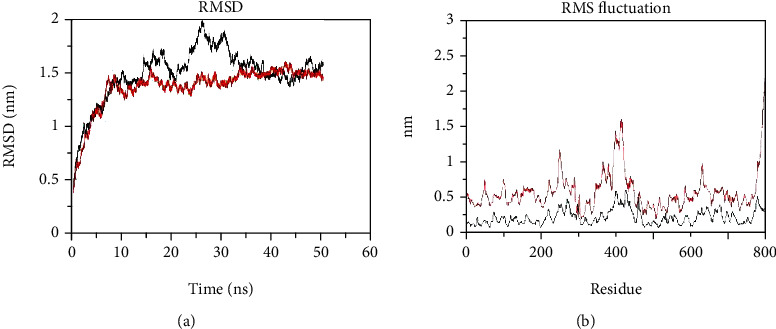
GROMACS analysis of backbone RMSD and RMSF as a function of time for the Gly482Ser variant and native at 50 ns molecular dynamics simulation (red: variant, black: native): (a) RMSD and (b) RMSF.

MDS have been widely applied to explore the structural consequences of the deleterious predicted SNPs. The results obtained from the above analysis conducted prompted us to further explore the dynamic behavior of the Gly482Ser variant and the native protein. We analyzed the root-mean-square deviation (RMSD), root-mean-square fluctuation (RMSF), radius of gyration (Rg), solvent accessible surface area (SASA), number of hydrogen bonds (NH), and secondary structure variation (DSSP between the Gly482Ser variant and the native protein). RMSD was plotted to examine the stability of the native protein to compare it with the stability of the variant protein [61]. The C*α*-RMSD is also a central origin to compute the protein system [62]. RMSD of WT and Gly482Ser variant started from 0.26 nm and increased to 1.47 nm in 8 ns, and at the end, the two plots came close together. The RMSD value at the end gets to 1.48 nm in WT and 1.36 nm in the Gly482Ser variant, and the RMSD plot of the Gly482Ser variant was more balanced than WT (Figure 2(a)). This variant decreased the flexibility of the PPARGC1A protein and had less flexibility than WT. The C*α*-RMSF was plotted to analyze the native protein flexibility and to compare it with the Gly482Ser variant protein's flexibility [61] (Figure 2(b)).

**Figure 3:**
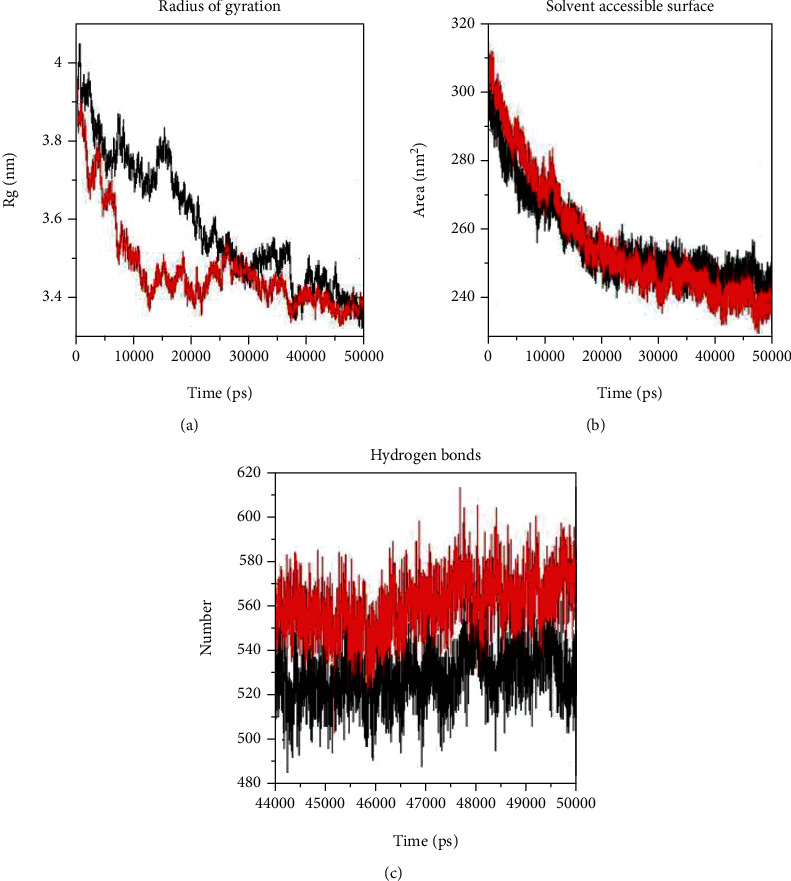
GROMACS analysis of Rg, SASA, and intramolecular hydrogen bonds of C*α* atoms for the Gly482Ser variant and in the PPARGC1A protein at 300 K: (a) Rg, (b) SASA, and (c) intramolecular hydrogen bonds. Variant was shown in black and native in red.

Rg is an indicator of the level of structure compaction, i.e., the polypeptide is unfolded or folded, causing it to gain and lose intramolecular H-bonds [63]. The competence, shape, and folding of the overall PPARGC1A structure at different time points during the trajectory can be seen in the plot of Rg. The results showed the more compact structure for the Gly482Ser variant with 3.47 nm, but the Rg value for WT was 3.58 nm (Figure 3(a)). The solvent-accessible surface area (SASA) was monitored throughout all simulations to measure the hydrophobic core's compactness. The SASA value for WT was 243 nm^2^ and for Gly482Ser variant was 237 nm^2^. The difference is not negligible. The Gly482Ser variant had less solvent-accessible surface area compared to WT and also had a more compact structure (Figure 3(b)). Hydrogen bonding maintains the conformation of a protein [63]. The number of hydrogen bonds was calculated as 521 for WT and 571 for the Gly482Ser variant. The Gly482Ser variant had higher hydrogen binding than WT (Figure 3(c)), indicating more intramolecular H-bonds that might result in a more rigid structure.

**Figure 4:**
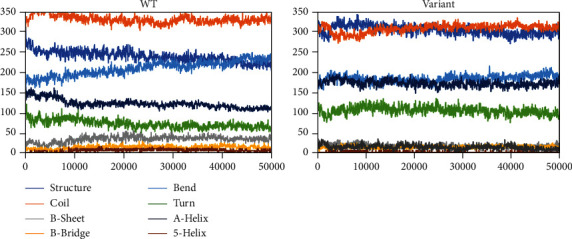
Secondary structural elements for the Gly482Ser variant and native in the PPARGC1A protein. Color of each secondary structure was displayed in legend.

Additional information on the flexibility of the Gly482Ser PPARGC1A variant was obtained by analyzing the time-course of change in the secondary structures of the native and Gly482Ser variant during 50 ns MDS using the DSSP program (Figure 4). The Gly482Ser variant had more secondary structures and tended to have a conserved secondary structure. The Gly482Ser variant showed an increase of *β*-sheet and coil and a decrease of turn than WT that both alterations decrease flexibility (Table 2). This suggests that the observed change in PPARGC1A due to this polymorphism is more related to structural changes.

### 3.5. Essential Dynamics

**Figure 5:**
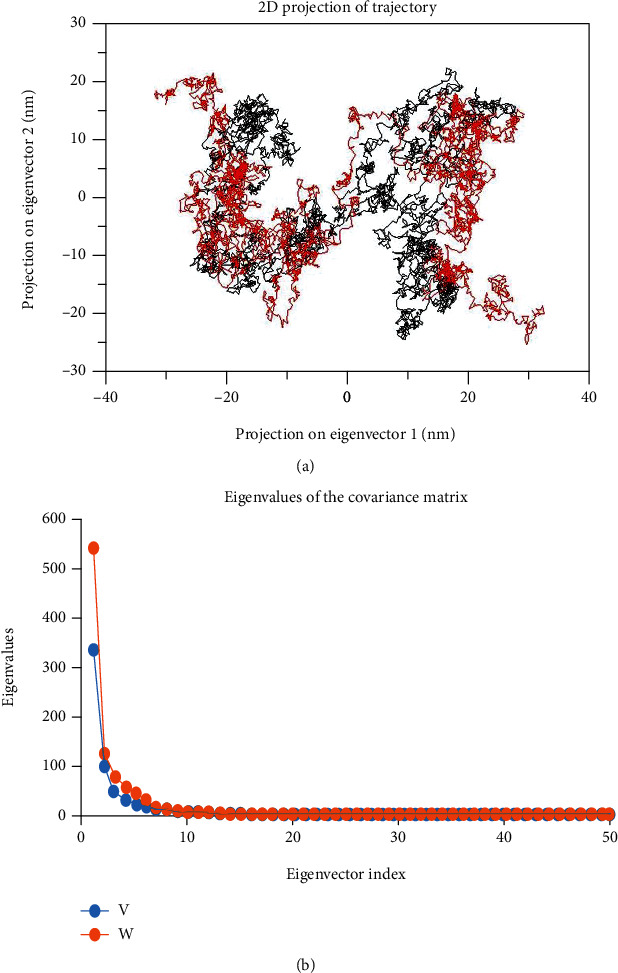
Principal component analysis. (a) Projection of the motion for wild type (black) and variant (red) in phase space along the PC1 and PC2. (b) Eigenvalues vs. eigenvector index plot was plotted for the first 50 eigenvectors, wild type (red) and variant (blue).

The dynamics of the Gly482Ser variant and native proteins were obtained through a principal component analysis (PCA) [64]. The projection of trajectories of the Gly482Ser variant and native proteins during the molecular dynamic's simulation in the phase space along the first two principal components (PC1, PC2) at 300 K is plotted in Figure 5. It predicts the large-scale collective motions for the Gly482Ser variant and native of the PPARGC1A protein. PCA analysis showed that, due to mutation, the structural dynamics is changing. The plot in Figure 5(a) clearly shows that compared to the native, the Gly482Ser variant occupied less space in the phase space while the native occupied more space. The first 50 eigenvectors were selected to compute concerted motions (Figure 5(b)). The eigenvalues were obtained from the diagonalization of the covariance matrix of atomic fluctuations. These results verify the overall increased flexibility of the native over the Gly482Ser variant. We conclude that the Gly482Ser variant causes the rigidity. The PCA analysis results agree with the results from molecular docking and MDS.

## 4. Discussion

PGC-1*α* has many functions as a transcriptional coactivator of PPAR-*γ* such as regulating lipid and energy metabolism [65]. Some studies regarding PGC-1*α* indicate that overexpression of PGC-1*α* reduces the accumulation of reactive oxygen species (ROS) and decrease apoptosis. It plays a protective role during oxidative stress connected at the transcriptional level to the upregulation of the mitochondrial antioxidant defense system by PGC-1*α*. PGC-1*α* also regulates the metabolism of lipids and carbohydrates and balances the consumption and storage of energy [6]. Insufficiency of this protein increases lipogenesis and hepatic steatosis. PGC-1*α* and PPAR-*α* also interact with each other. PPAR-*α* regulates the oxidation of fatty acids in fasting. This protein is mainly expresses in the liver [65]. According to reports, in patients with type 2 insulin-resistant diabetes, the PGC-1*α* responsive genes were downregulated [66]. In Patti et al.'s investigation on diabetic Mexican-American, there was a correlation between insulin resistance type 2 diabetes and reducing PGC-1*α* in the skeletal muscle [67].

**Figure 6:**
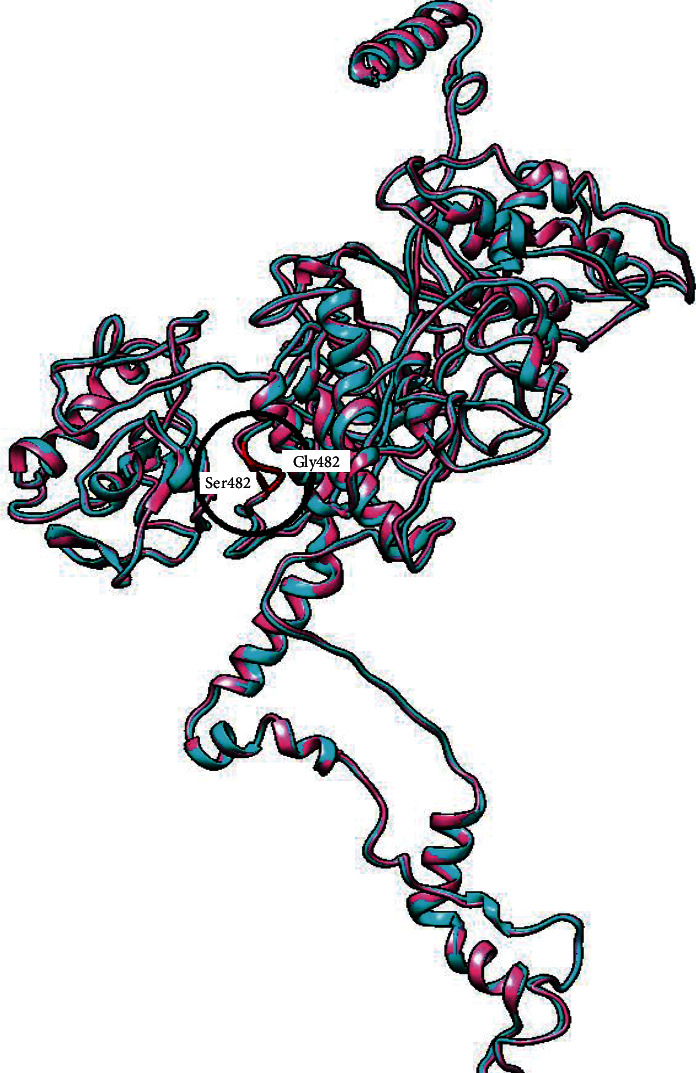
Superimposition of secondary structures of the Gly482Ser variant and native by UCSF Chimera (Gly482 (cyan) : Ser482 (pink)). The variation site was highlighted in red.

nsSNPs are SNPs that may alter protein conformation and function and result in pathogenic phenotypes. Among all PPARGC1A polymorphisms, the Gly482Ser variant has been the most studied [68]. Its correlation with the risk of type2 diabetes was reported in many studies. We performed a superimposition of the Gly482Ser variant PPARGC1A and wild type using UCSF Chimera tool [69] to explore the effect of the Gly482Ser variant on the PPARGC1A structure. We gained 0.633 RMSD, showing that the Gly482Ser variant has a remarkable impact on the PPARGC1A structure (Figure 6).

This polymorphism probably triggers NAFLD and CAD's pathogenesis in type 2 diabetic patients by altering the PGC-1*α* interaction with other transcription factors and affecting oxidative stress and lipid metabolism [6, 70]. It has also been suggested that Gly482Ser polymorphism correlates with an increased risk of insulin resistance, obesity, and type 2 diabetes [20, 70, 71]. In one study, Lai and colleagues investigated the association among the PPARGC1A variations, including Gly482Ser, with DNA damage, diabetes, and cardiovascular diseases. Their research showed that the PPARGC1A variant has significant association with DNA damages and these diseases [72]. In another study, Yongsakulchai et al. suggested that the combination of PPAR-*γ* C1431T, PGC-1*α* Gly482Ser, and LXR*α*−115G/A polymorphisms increase the risk of CAD and were predictive of the severity of coronary atherosclerosis [73]. We studied the in vitro effect of Gly482Ser polymorphism on NAFLD and CAD's pathogenesis in type 2 diabetic patients in two of our previous studies [26, 27].

In the present study, we used SNP prediction tools and found that the Gly482Ser variant causes instability of PPARGC1A. Molecular docking shows that the native protein has a ZDOCK score higher than the variant, indicating that the substitution of Gly for Ser disturbs the interaction between PPARGC1A and PPAR-*γ* and causes disease and deterioration in protein stabilization. Apparent instability and loss flexibility were mostly seen in the RMSF, RMSD, and SASA plots that were accompanied by a significant number of intramolecular NH bonds for Gly482Ser when compared to native PPARGC1A, and with an increase of *β*-sheet and coil and decrease of turn in the DSSP plot of the Gly482Ser variant.

We also found a relatively lower Rg value, which associates with a reduction in the Gly482Ser variant protein stability. These results also correspond with the number of intramolecular H-bonds and the results of DSSP. More intramolecular NH bonds in the Gly482Ser variant structure might help its rigidity and make it nonflexible. This might be due to the conserved secondary structure of the variant which affects protein folding and might disorganize the structural conformation and catalytic function of the protein structure, as well as induce T2DM and NAFLD and CAD. Therefore, we suggest that the Gly482Ser variant has a significant impact on protein function.

This study was further extended by analyzing PCA for the Gly482Ser variant and native. The PCA analysis was carried out to understand the collective residual motion of the Gly482Ser variant and native. We observed a difference in conformational changes in motions of C*α* atoms of the Gly482Ser variant in comparison with the wild type. This may significantly impact the structural conformation of the protein which also affects the function of the PPARGC1A protein. Because this polymorphism made the PPARGC1A structure more rigid, Gly482Ser polymorphism has a critical damaging impact on protein function and its structural orientation. This prediction is supported by the cited experimental data [26, 27], and the results of this study will help wet lab researchers expand potent treatments against PPARGC1A.

## 5. Conclusion

The present study is an in-depth computational study into the genotype–phenotype association of the deleterious Gly482Ser variant in the PPARGC1A. We provided evidences of damaging conformational changes in the PPARGC1A protein that have a notable role in inducing disease-associated phenotypes. We showed that the Gly482Ser variant in the PPARGC1A protein can probably produce a disease-associated phenotypic effect by having a major impact on the structural conformation of the PPARGC1A protein.

The stability of a protein is required for its correct function [13, 74–77]. The stability prediction tools found that this variant decreases/destabilizes the PPARGC1A. Molecular docking and molecular dynamics simulation indicated the Gly482Ser variant has deleterious effects on the structural and functional attributes of PPARGC1A, resulting in functional and structural alterations in the PPARGC1A. The analysis highlights the difference in the dynamics of PPARGC1A variants. The dynamics of protein are dependent on the structural flexibility of PPARGC1A [78], and H-bonds are essential to stabilize the protein structure [79]. All MD results displayed the increased compactness of the Gly482Ser variant structure. The PCA analysis also showed that, due to mutation, the structures change their original geometry. This may produce a major impact on the structural conformation of the PPARGC1A protein, resulting in the loss of the protein function. This suggests that the Gly482Ser variant protein is more compact or rigid than the native protein and that this might result in decreasing the PPARGC1A expression in patients with related diseases. Due to the Gly482Ser variant, the structure becomes more rigid and compact at the same time that it needs to fulfill its native function of a maintaining proper conformational geometry of the protein. The results of this study elucidate the role of Gly482Ser polymorphism in PPARGC1A and may provide useful information for the design of PPARGC1A variant-based therapeutic strategies against CAD, NAFLD, T2DM, obesity, hypertension, and metabolic diseases.

## Figures and Tables

**Table 1:** SNP tools for the stability prediction of the Gly482Ser variant with DDG.

Computational algorithms	Prediction
I-Mutant2.0 DGG (kcal/mol)	Decreases stability -0.83
MUpro DGG (kcal/mol)	Decreases stability -0.32
DUET DGG (kcal/mol)	Destabilizing -0.99
DGG (kcal/mol) DynaMut	Destabilizing -0.84
mCSM DGG (kcal/mol)	Destabilizing -0.99
SDM DGG (kcal/mol)	Destabilizing -3.33
CUPSAT PANTHER	Destabilizing Probably damaging

**Table 2:** Secondary structure percentage the Gly482Ser variant and native.

DSSP	Structure	Coil	*β*-Sheet	*β*-Bridge	Bend	Turn	*α*-Helix	5-Helix	3-Helix
Variant	0.30	0.42	0.04	0.02	0.26	0.09	0.15	0.01	0.01
Native	0.38	0.38	0.02	0.02	0.23	0.13	0.21	0.00	0.02

## Data Availability

We state that our data is available during the study.
